# Supplementary material for: Early life adversity in male mice sculpts reward circuits
Source: Neurobiol Stress. 2021 Oct 16;15:100409. doi: 10.1016/j.ynstr.2021.100409 (PMC8554344; doi:10.1016/j.ynstr.2021.100409)
Supplement: Multimedia component 1 [file mmc1.docx]

**Early Life Adversity in Male Mice Sculpts Reward Circuits.**

Kara M. Wendel1*, Annabel K. Short,2 Brenda P. Noarbe,3 Elizabeth Haddad,2 Anton M. Palma,3 Michael A. Yassa,4 Tallie Z. Baram,1,2 Andre Obenaus1,2

**Supplemental Materials**

1. Supplemental Figure 1
2. Supplemental Figure 2
3. Supplemental Figure 3
4. Supplemental Table 1


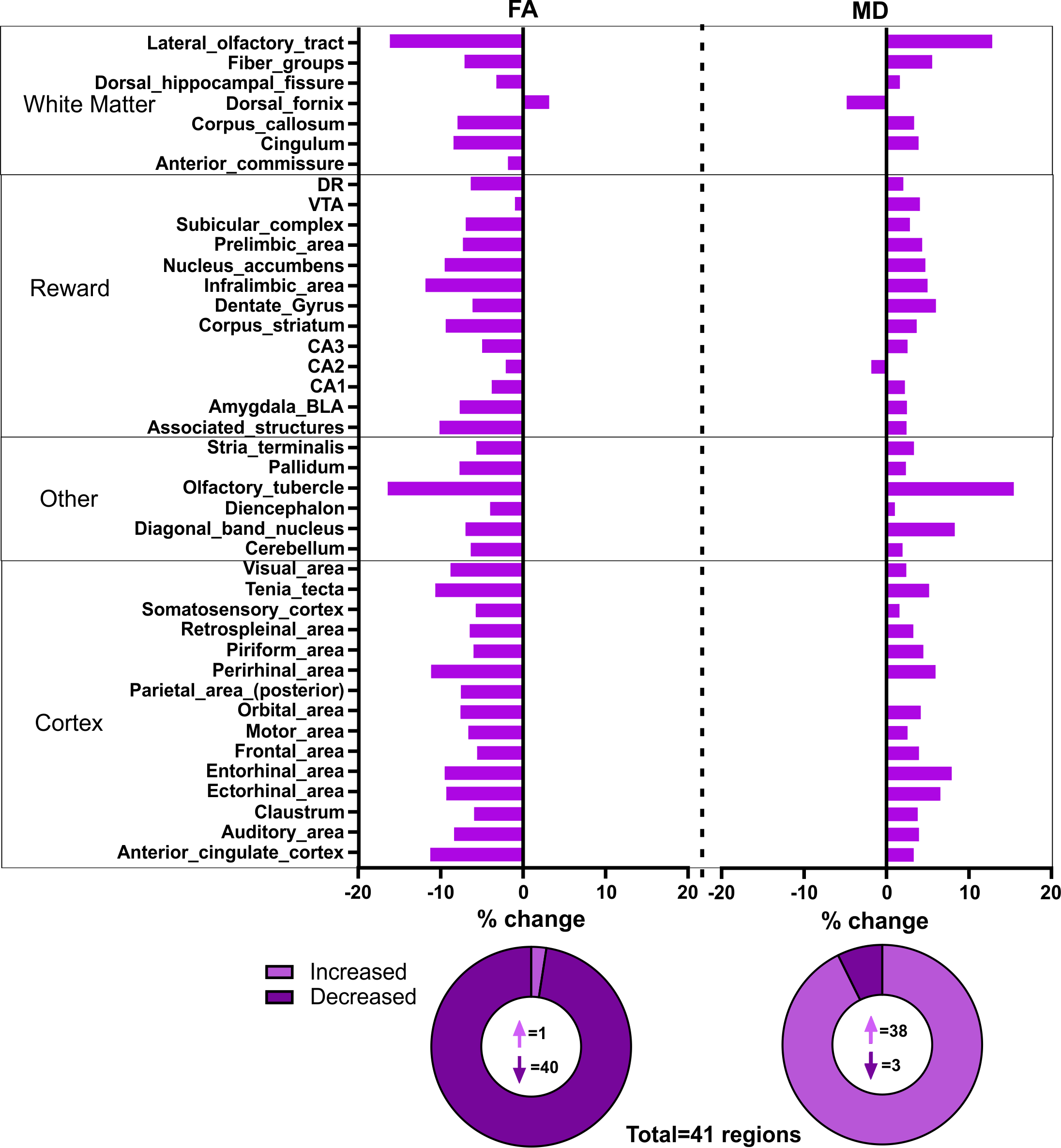


**Supplemental Figure 1. FA and MD also exhibited brain wide regional changes after ELA.** Percent change in FA and MD of the ELA mice compared to controls also showed that many regions were altered after ELA. FA exhibited widespread decreases across almost all regions after ELA, whereas MD showed increases across majority of the regions following ELA. Pie charts show the distribution of regions with percent change that were increased versus decreased for the ELA group compared to controls.


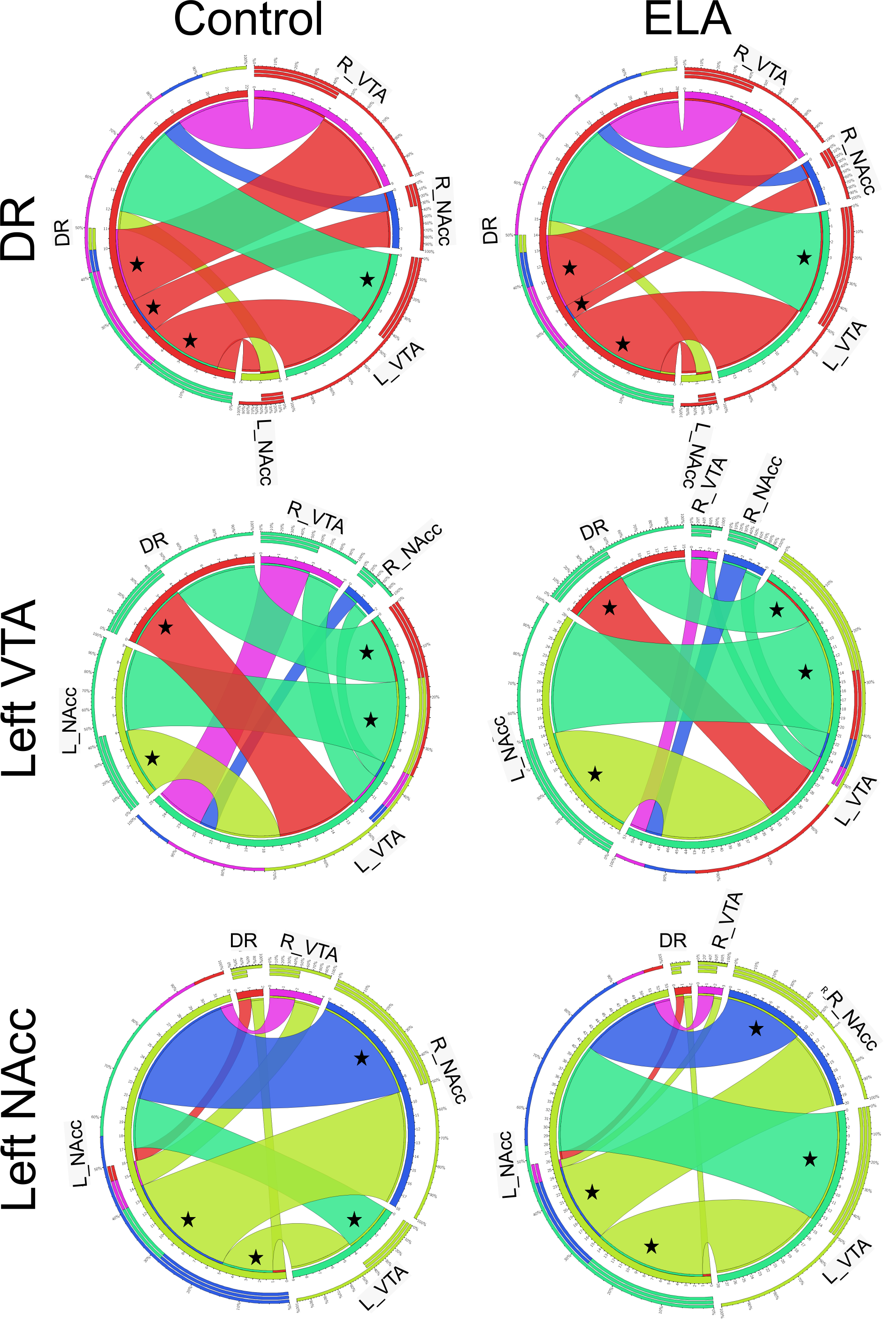


**Supplemental Figure 2. Connectivity of the DR, VTA, and NAcc was altered in ELA mice.** The connectivity of each region highlights the alterations after ELA (*). The ELA group exhibited a stronger connectivity between the DR and the left VTA, and decreased connectivity between DR and the right NAcc (*). The left VTA connectivity was increased to the NAcc, but conversely decreased connectivity to the DR in the ELA group. In the ELA group, the left NAcc had increased connectivity to the left VTA but decreased connectivity to the left and right NAcc.


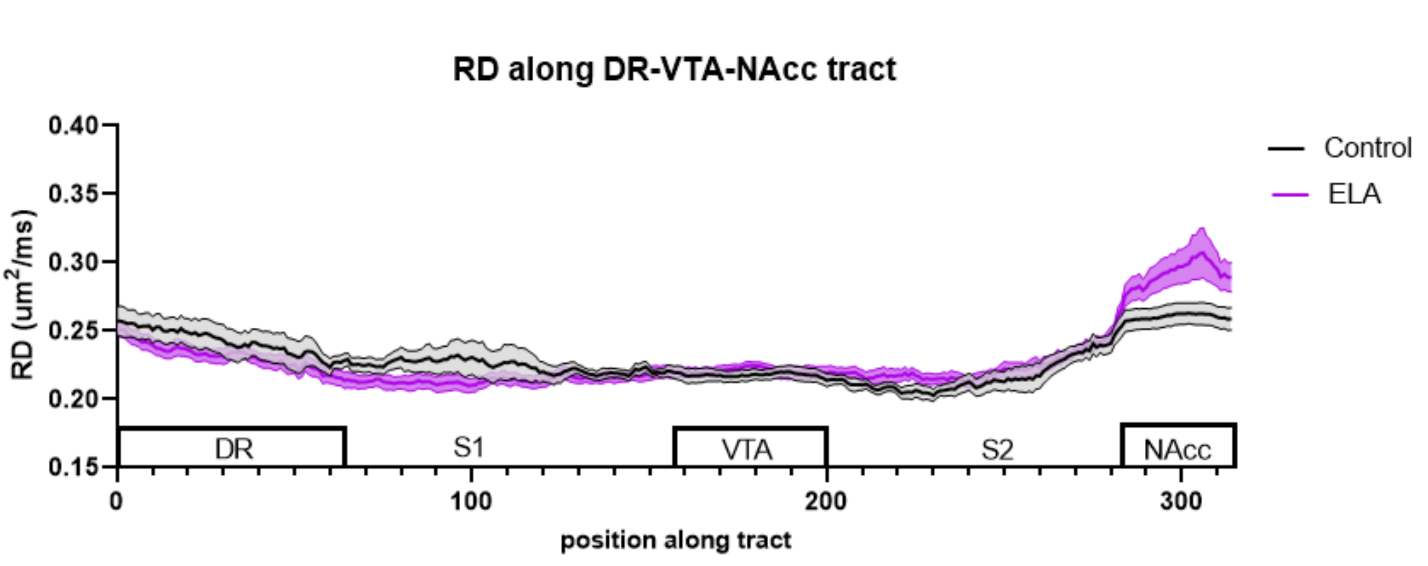


**Supplemental Figure 3.** Radial diffusivity (RD) did not report significant changes within the DR-VTA-NAcc tract. The RD of the tract showed no changes throughout the DR, S1 and VTA segments of both control and ELA groups. ELA mice exhibited a small decrease in RD within the S1, followed by a modest increase in RD within the S2. RD was increased upon entry into the NAcc.


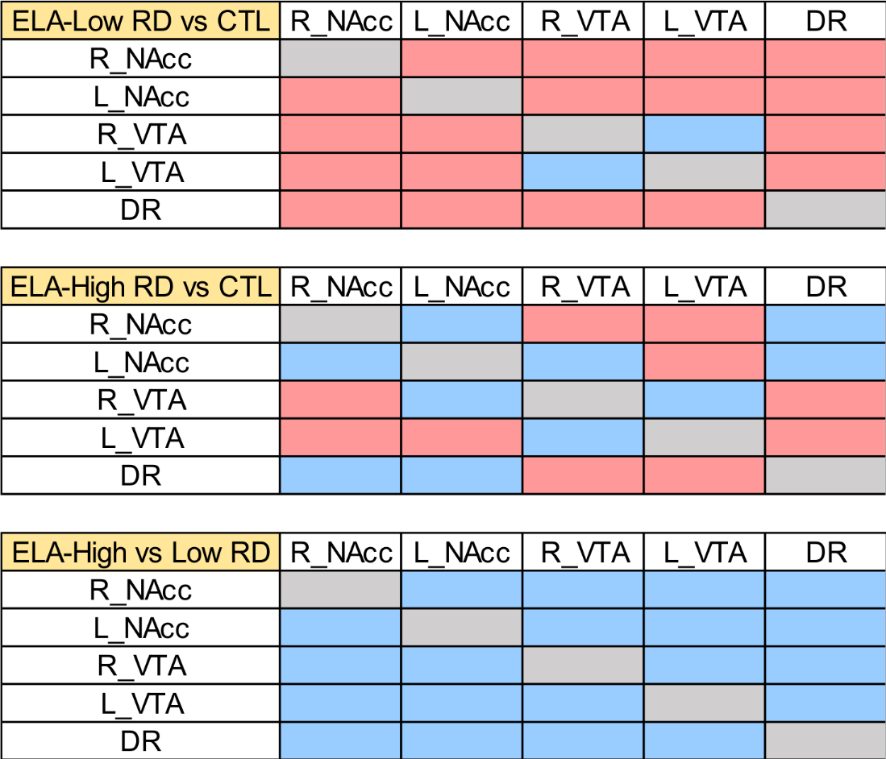


**Supplemental Table 1.** Heat maps of reward circuit connectivity. Relative changes in streamline numbers of DR-VTA-NAcc connectivity of ELA mice compared to controls. (Red = increase, blue = decrease, grey = no change).
